# A porcine placental extract prevents steatohepatitis by suppressing activation of macrophages and stellate cells in mice

**DOI:** 10.18632/oncotarget.24587

**Published:** 2018-02-27

**Authors:** Liang Xu, Naoto Nagata, Mayumi Nagashimada, Fen Zhuge, Yinhua Ni, Guanliang Chen, Junzo Kamei, Hiroki Ishikawa, Yasuhiko Komatsu, Shuichi Kaneko, Tsuguhito Ota

**Affiliations:** ^1^ Department of Cell Metabolism and Nutrition, Advanced Preventive Medical Sciences Research Center, Kanazawa University, Kanazawa, Japan; ^2^ Department of Pathophysiology and Therapeutics, Hoshi University School of Pharmacy and Pharmaceutical Sciences, Tokyo, Japan; ^3^ Snowden Co., Ltd, Tokyo, Japan; ^4^ Division of Metabolism and Biosystemic Science, Department of Medicine, Asahikawa Medical University, Asahikawa, Japan

**Keywords:** placental extract, steatohepatitis, macrophage polarization, inflammation, oxidative stress

## Abstract

Nonalcoholic fatty liver disease (NAFLD) is caused by ectopic fat accumulation in the liver. NAFLD is associated with hepatic inflammation and oxidative stress, resulting in nonalcoholic steatohepatitis (NASH) with advanced fibrosis. Placental extracts have been used to treat various chronic diseases due to their antioxidative effect. However, the effects of the extracts on the development of NASH have yet to be elucidated. Here, we demonstrated that supplementation with an oral porcine placental extract (PPE) attenuated lipid accumulation and peroxidation, insulin resistance, inflammatory and stress signaling, and fibrogenesis in the liver of NASH model mice fed a high-cholesterol and high-fat diet. The PPE reduced the number of M1-like liver macrophages, but increased the number of anti-inflammatory M2-like macrophages, resulting in a predominance of M2 over M1 macrophage populations in the liver of NASH mice. Accordingly, the PPE suppressed lipopolysaccharide-induced M1 polarization in isolated murine peritoneal macrophages, whereas it facilitated interleukin 4-induced M2 polarization. Furthermore, the PPE reduced the hepatic stellate cell (HSC) activation associated with the attenuated transforming growth factor-β/Smad3 signaling, both in the liver of NASH mice and in RI-T cells, a HSC line. The PPE may be a potential approach to prevent NASH by limiting lipid peroxidation, promoting M2 macrophage polarization, and attenuating HSC activation.

## INTRODUCTION

Nonalcoholic fatty liver disease (NAFLD) is frequently described as a spectrum of diseases from simple steatosis to nonalcoholic steatohepatitis (NASH) with advanced fibrosis [1]. A “multiple-parallel-hit” theory has been proposed in which dysregulated lipid metabolism and insulin resistance are the “first-hit” to the liver, followed by a “second-hit”, or “multi-hits”, such as oxidative stress and pro-inflammatory chemokines and cytokines [2, 3]. Along with the development of NASH, oxidative stress and pro-inflammatory cytokines activate immune cells, such as liver macrophages (i.e., Kupffer cells) and T-lymphocytes. Activation of these immune cells results in chronic inflammation and insulin resistance in the liver [4–8]. Our previous study developed a cholesterol- and saturated fatty acid-induced mouse model of lipotoxic NASH, replicating the pathophysiological features of human NASH [8, 9]. The liver of this model mouse exhibits excessive lipid accumulation and aberrant activation of liver macrophages and hepatic stellate cells (HSCs), resulting in the exacerbation of hepatic insulin resistance, inflammation, and fibrosis [9].

To date several pharmacological agents, such as metformin [10], thiazolidinediones [11], vitamin E [12], and carotenoids [13, 14] have been tested as treatments for NASH. However, these agents are generally insufficient to ameliorate liver inflammation and fibrosis, and have raised safety concerns. Therefore, a potential therapy with minimal adverse effects has been eagerly awaited. Placental extracts prepared from human or animal placenta have been used as ingredients in medicines, health foods, and cosmetics. Orally administered placental extracts have been reported to be effective for menopausal disorders [15, 16] and their symptoms (e.g., knee pain, shoulder stiffness, and wrinkles below the eyes) [17–19]. Togashi et al. reported that an orally administered human placental extract inhibits alcohol-induced hepatic disorder in a mouse model [20, 21]. Moreover, recent clinical studies have reported that a human placental extract decreases plasma levels of alanine aminotransferase (ALT) and aspartate aminotransferase (AST) and prevents the progression of alcoholic hepatitis and NASH in human patients [22, 23]. However, the underlying mechanisms of action of placental extracts have not been fully elucidated.

Porcine placental extract (PPE) have been developed as an alternative source to human placental extracts due to biosafety concerns [17–19, 24]. In the current study, we examined PPE-mediated mitigation of oxidative stress, insulin resistance, lipid accumulation, chronic inflammation, and fibrosis in the liver of a diet-induced NASH mouse model.

## RESULTS

### The PPE alleviates hepatic steatosis and oxidative stress in NASH mice

**Figure 1:**
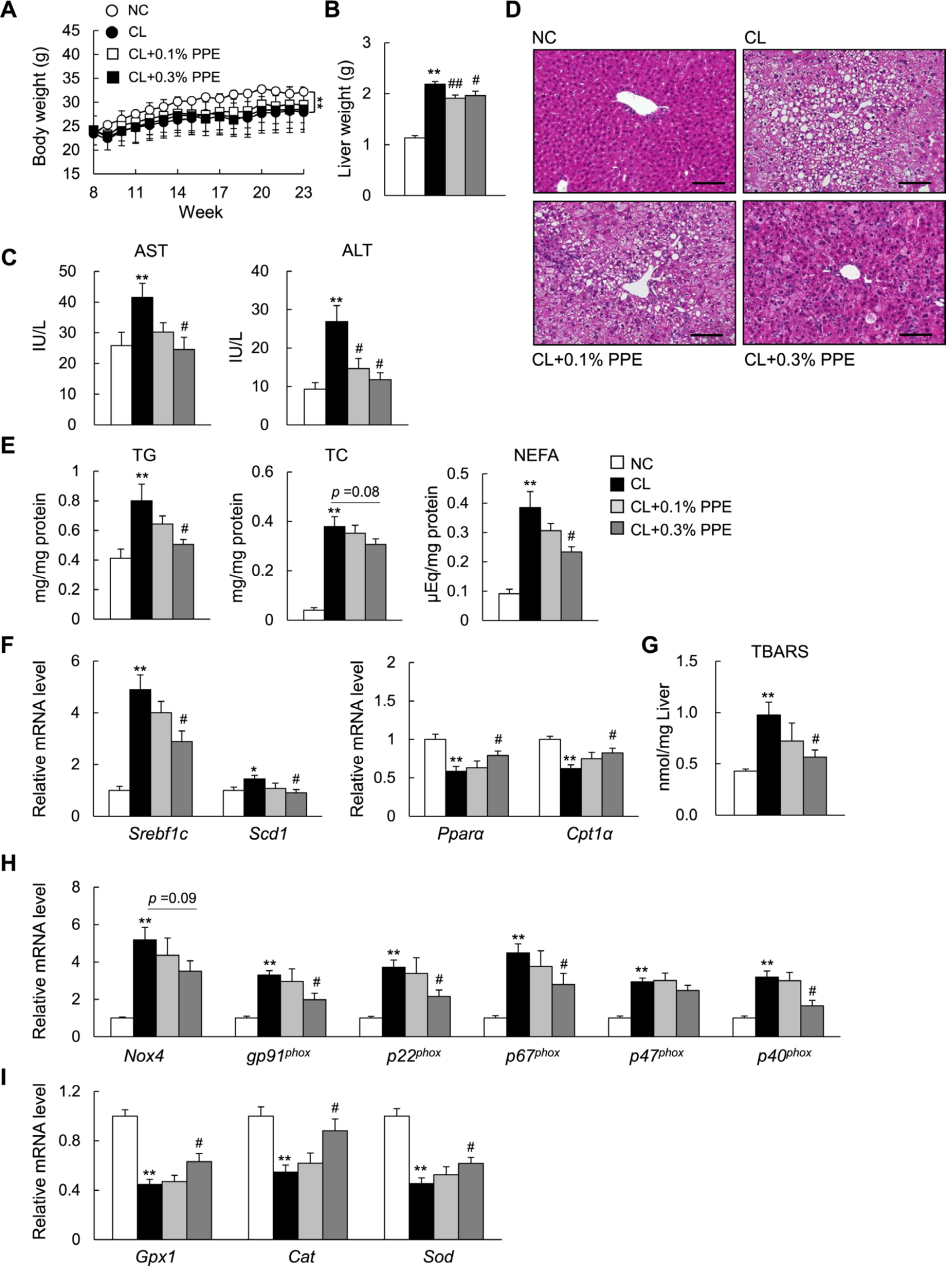
Porcine placental extract (PPE) supplementation attenuates hepatic steatosis in CL diet-fed mice (**A**) Body weights of the mice. (**B**) Liver weights of the mice. (**C**) Levels of plasma aspartate aminotransferase (AST) and alanine aminotransferase (ALT). (**D**) Representative hematoxylin and eosin-stained liver section. Scale bars = 100 μm. (**E**) Levels of hepatic triglycerides (TG), total cholesterol (TC), and non-esterified fatty acids (NEFA). (**F**) Quantitative real-time PCR of hepatic mRNA expression of genes involved in fatty acid synthesis and β-oxidation. (**G**) Levels of hepatic thiobarbituric acid reactive substances (TBARS). (**H**) Hepatic mRNA expression of NADPH oxidase complex. (**I**) Hepatic mRNA expression of antioxidant genes. *n* = 6–8. ^*^P < 0.05, ^**^P < 0.01 vs. normal chow (NC) diet-fed mice. ^#^*P* < 0.05, ^##^*P* < 0.01, vs. CL diet-fed mice.

A high-fat, high-cholesterol, and cholate (CL) diet induces hepatic steatosis and inflammation, eventually leading to steatohepatitis in C57BL/6J mice similar to that of human NASH [8, 9]. To determine the effect of the PPE on diet-induced NASH, we fed C57BL/6J mice with normal chow (NC), the CL diet, or the CL diet supplemented with 0.1% (w/w) or 0.3% (w/w) PPE for 15 weeks. The CL diet significantly increased liver weight (Figure 1B) and plasma AST and ALT levels (Figure 1C). PPE supplementation attenuated the increase in liver weight caused by the CL diet (Figure 1B), without reducing body weight (Figure 1A) or food intake (Table 1). Additionally, the PPE decreased plasma AST and ALT levels dose-dependently, suggesting alleviation of CL diet-induced liver damage (Figure 1C). In parallel, the PPE significantly attenuated hepatic steatosis (Figure 1D), and the increase in liver triglyceride (TG) and non-esterified fatty acid (NEFA) levels caused by the CL diet (Figure 1E), although plasma TG, total cholesterol (TC), and NEFA levels were not decreased (Table 1). The reduction in hepatic steatosis was accompanied by decreased expression of the lipogenic genes sterol regulatory element binding transcription factor 1c (*Srebf1c*) and stearoyl-CoA desaturase 1 (*Scd1*) (Figure 1F). Moreover, the PPE restored the decreased expression of genes involved in mitochondrial fatty acid β-oxidation (*Pparα* and *Cpt1α*) in the liver of CL-diet fed mice (Figure 1F).

**Table 1:** Metabolic parameters after 15 weeks of feeding

	NC	CL	CL + 0.1% PPE	CL + 0.3% PPE
**Food intake (Kcal/day)**	12.3 ± 0.3	11.1 ± 0.4^*^	13.0 ± 0.3^#^	12.9 ± 0.5^##^
**Plasma TG (mg/dL)**	36.7 ± 3.8	14.9 ± 2.2^*^	12.8 ± 4.0	12.8 ± 3.0
**Plasma TC (mg/dL)**	65.2 ± 4.2	97.7 ± 3.0^**^	97.3 ± 11.6	101.7 ± 9.9
**Plasma NEFA (mmol/L)**	1.7 ± 0.1	0.9 ± 0.1^**^	0.8 ± 0.1	0.7 ± 0.1
**Plasma TBARS (nmol/mL)**	1.6 ± 0.9	2.2 ± 0.2^**^	2.0 ± 0.2	1.4 ± 0.1^#^
**Plasma IL-6 (pg/mL)**	1.4 ± 0.3	8.9 ± 0.8^**^	4.9 ± 0.3^#^	5.2 ± 1.2^#^
**Plasma CCL-2 (pg/mL)**	8.7 ± 1.1	25.7 ± 4.1^**^	18.9 ± 3.4	14.4 ± 1.8^#^

Food intake was determined at the middle period of PPE treatment. Plasma parameters data were obtained from 23-week-old mice on different diets. For measuring the plasma metabolic parameters, the fasting blood was collected from the mice fasted for 16 hours. Data are presented as means ± SEM (*n =* 6–8). ^*^P < 0.05, ^**^P < 0.01 vs. NC diet-fed mice. ^#^*P* < 0.05, ^##^*P* < 0.01, vs. CL diet-fed mice.

### The PPE decreases lipid accumulation in primary hepatocytes

To examine whether the PPE directly attenuates lipid accumulation in hepatocytes, we incubated primary hepatocytes with oleate in the presence or absence of the PPE. The PPE treatment decreased lipid accumulation, as assessed by Oil red O staining, in oleate-loaded hepatocytes (Supplementary Figure 1A). The reduction in lipid accumulation was accompanied by suppression of lipogenic gene (*Srebf1c* and *Fas*) expression, and by increased expression of genes involved in fatty acid oxidation (*Pparα, Cpt1α, Lcad*, and *Acox1*) and lipid transport (*Lcat*, *Apob*, and *Mttp*) in oleate-treated hepatocytes (Supplementary Figure 1B). Furthermore, to evaluate the effect of the PPE on oxidative stress in CL diet-fed mice, we determined the hepatic and plasma levels of thiobarbituric acid reactive substances (TBARS), a marker of lipid peroxidation. The PPE decreased lipid peroxidation (Table 1 and Figure 1G) and decreased hepatic gene expression of the NADPH oxidase subunits (Figure 1H), whereas it increased expression of antioxidative stress genes in the liver of NASH mice (Figure 1I).

### The PPE restores glucose intolerance and insulin sensitivity in NASH mice

**Figure 2:**
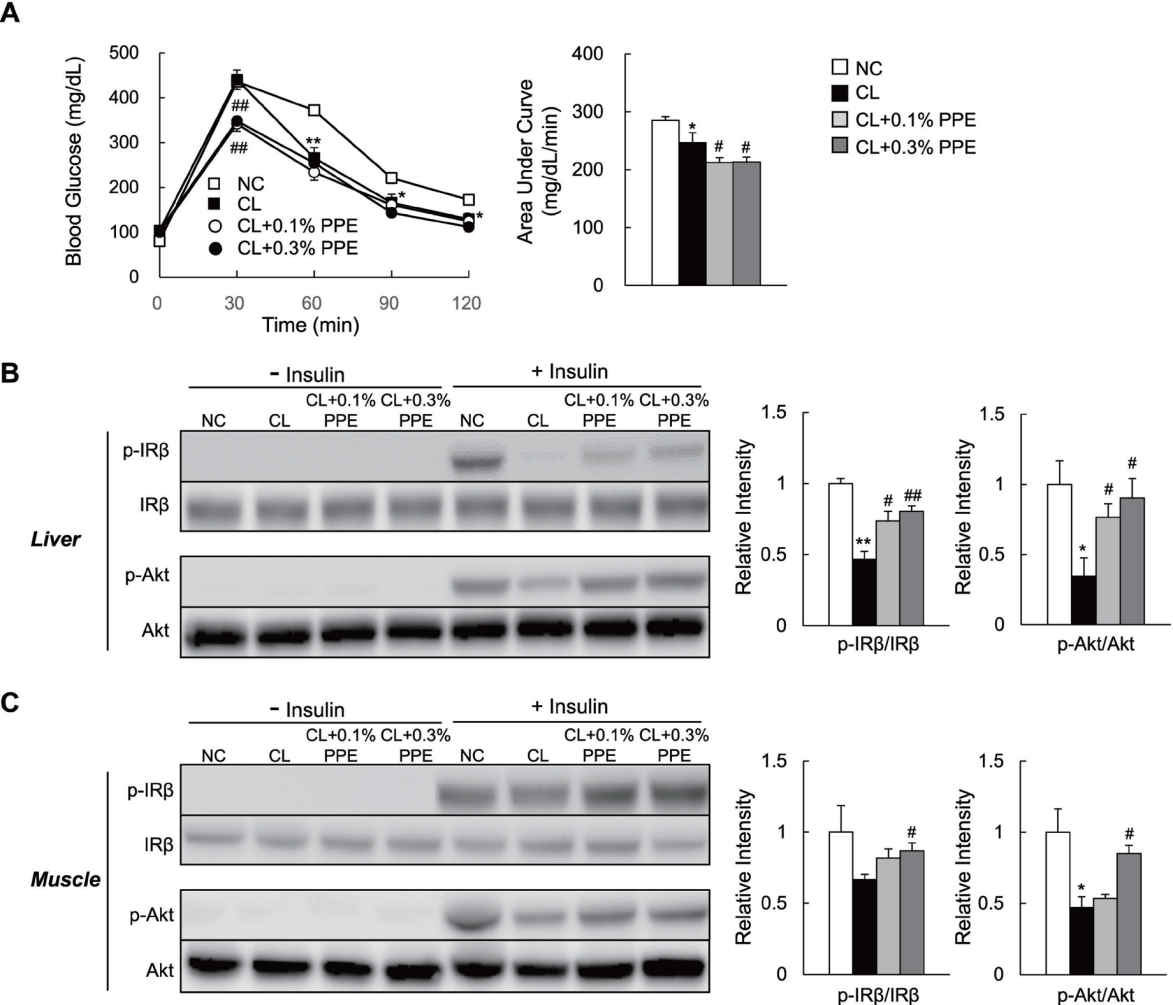
PPE supplementation ameliorates glucose intolerance and insulin sensitivity in CL diet-fed mice (**A**) Glucose tolerance test (GTT; 2 g/kg body weight) after 14 weeks (*n* = 6–8/group). Area under the curve calculations. (**B, C**). Mice on the indicated diet for 15 weeks were injected in the tail vein with saline (*n* = 3/group) or insulin (*n* = 4/group, 5 U/kg body weight), and killed 7 min after the injection. Total liver and quadriceps muscle lysates were immunoblotted for p-IRβ, IRβ, p-Akt, and Akt, quantitated, and presented as means ± SEM. ^*^P < 0.05, ^**^P < 0.01 vs. NC diet-fed mice. ^#^*P* < 0.05, ^##^*P* < 0.01, vs. CL diet-fed mice.

Ectopic fat accumulation in the liver has been linked to the development of glucose intolerance and insulin resistance [1]. We performed the glucose tolerance test (GTT) to determine whether the PPE improves glucose metabolism in NASH mice. PPE supplementation improved glucose tolerance in CL diet-fed mice during the GTT (Figure 2A), although insulin secretion during the GTT was not decreased by the PPE (data not shown). We also performed biochemical studies to investigate the effect of the PPE on insulin sensitivity, and found that PPE administration restored insulin-stimulated tyrosine phosphorylation of insulin receptor β subunit (p-IRβ) and serine phosphorylation of Akt (p-Akt) in the liver and skeletal muscle of CL diet-fed mice (Figure 2B and 2C).

### The PPE attenuates inflammation and endoplasmic reticulum (ER) stress in the liver of NASH mice

**Figure 3:**
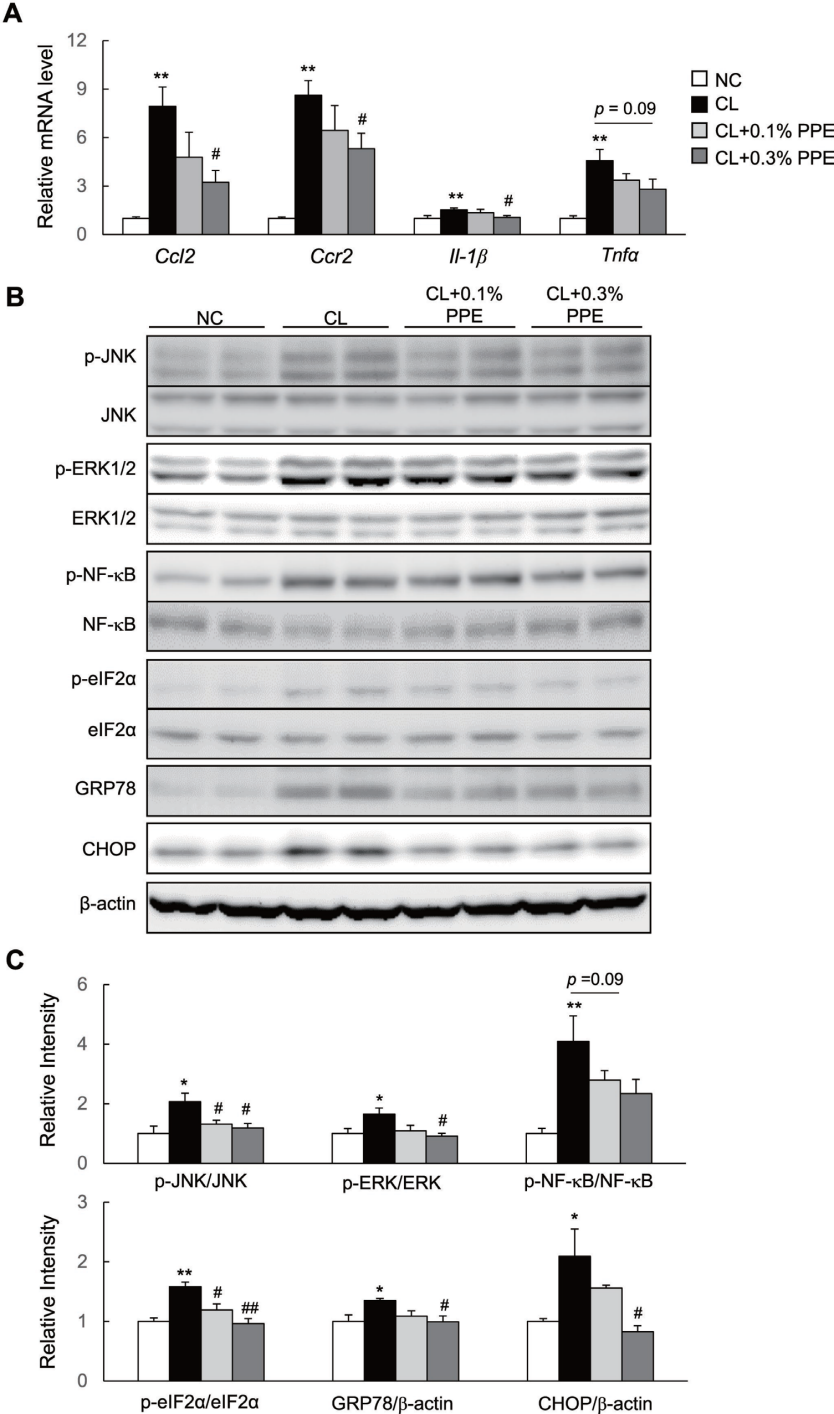
PPE attenuates hepatic inflammation in nonalcoholic steatohepatitis (NASH) mice (**A**) mRNA expression of *chemokine (C-C motif) ligand 2 (Ccl2), Ccl2 receptor (Ccr2), interleukin (Il)-1β, and tumor necrosis factor (Tnf)-α* in the liver. (**B**). Immunoblot of p-Jun N-terminal kinase (p-JNK), p-extracellular signal-regulated kinase (p-ERK), phosphorylated nuclear kappa beta (p-NF-κB), p-eIF2α, GRP78, and CHOP in the liver. (**C**) Quantification of p-JNK, p-ERK, p-NF-κB, p-eIF2α, GRP78, and CHOP in the liver. *n* = 6–8. ^*^P < 0.05, ^**^P < 0.01 vs. NC diet-fed mice. ^#^*P* < 0.05, ^##^*P* < 0.01, vs. CL diet-fed mice.

Chemokine (C-C motif) ligand 2 (Ccl2) increases recruitment of Ccl2 receptor (Ccr2)-positive inflammatory monocytes into the liver [25, 26]. These recruited cells produce large amounts of proinflammatory mediators and promote insulin resistance and NASH in mice [27]. Here, we found marked induction of *Ccl2*, *Ccr2*, and *interleukin* (IL)*-1β* in the liver of CL diet-fed mice, which decreased significantly in PPE-treated mice (Figure 3A). The PPE also tended to decrease mRNA expression of tumor necrosis factor (*Tnf*)*-α* (Figure 3A). Additionally, the PPE significantly suppressed CL diet-induced phosphorylation of c-Jun N-terminal kinase (JNK) and extracellular signal-regulated kinase (ERK), and tended to decrease levels of phosphorylated nuclear kappa beta (p-NF-κB) p65 in the CL + 0.3% PPE group, although this decrease was not significant (Figure 3B and 3C). Moreover, the PPE also decreased the protein levels of phosphorylated eIF2α, GRP78, and CHOP, which are involved in ER stress (Figure 3B and 3C). In accordance with attenuated hepatic inflammation, the plasma levels of IL-6 and CCL2 also decreased in response to PPE supplementation (Table 1).

### The PPE causes a predominance of M2 over M1 macrophage populations in the liver of NASH mice

**Figure 4:**
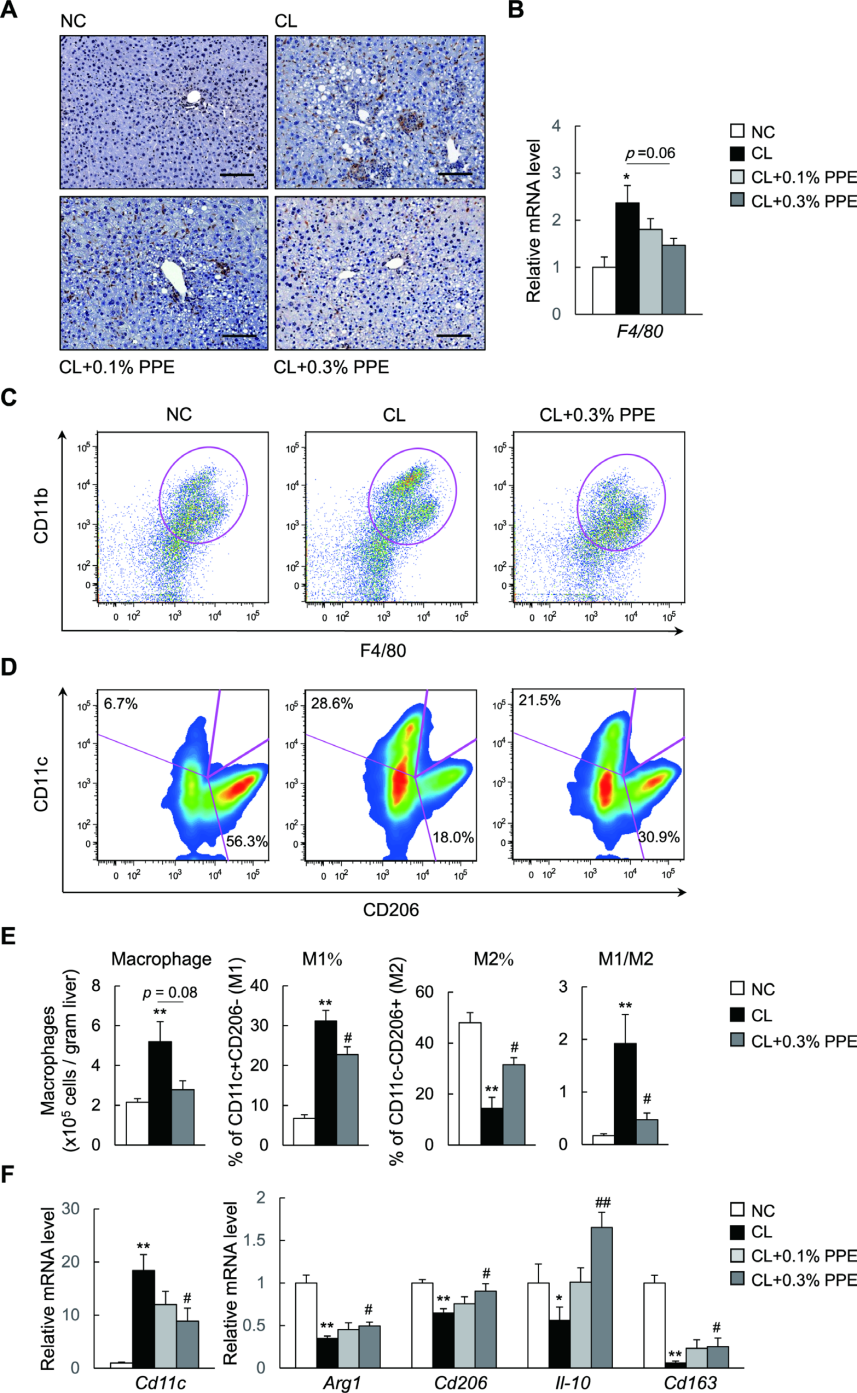
PPE promotes a predominance of M2 macrophage populations over M1 in the liver (**A**) Representative F4/80-stained liver section. Scale bars = 100 μm. (**B**) mRNA expression of *F4/80* in the liver. (**C**) Representative plot of total macrophages in the liver. (**D**) Frequency of M1, M2-type macrophages in the liver. (**E**) Quantitation of total macrophages, percentages of M1, M2-type macrophages and the M1/M2 macrophage ratios in the liver. (**F**) mRNA expression of *Cd11c* and M2 markers in the liver. ^*^P < 0.05, ^**^P < 0.01 vs. NC diet-fed mice. ^#^*P* < 0.05, ^##^*P* < 0.01, vs. CL diet-fed mice.

Significant improvements in chronic inflammation in the liver of PPE-treated NASH mice prompted us to investigate the effects of the PPE on the homeostasis of hepatic macrophages. Here, we found that the PPE tended to decrease the number of F4/80^+^-cells in the liver of NASH mice, as assessed by immunostaining (Figure 4A) and mRNA expression levels of the macrophage marker *F4/80* (Figure 4B). To quantify the numbers of total (CD45^+^CD11b^+^F4/80^+^), pro-inflammatory M1-like (CD11c^+^CD206^-^), and anti-inflammatory M2-like (CD11c^-^CD206^+^) macrophages in the liver, we analyzed hepatic immune cells by fluorescence-activated cell sorting (FACS). Compared with the NC group, the number of total macrophages and M1-like macrophages in the liver increased by 1.6-fold and 3.8-fold in the CL group, respectively, suggesting that the CL diet induced accumulation of pro-inflammatory macrophages (Figure 4C–4E). Although the PPE did not alter the total number of liver macrophages markedly, it decreased M1-like macrophage accumulation by 44.2% compared with that in the CL group (Figure 4D and 4E). In contrast, the PPE increased the number of M2-like liver macrophages by 1.4-fold, resulting in a predominant M2-like macrophage population (Figure 4D and 4E). In accordance with these findings, mRNA expression of the M1-like macrophage marker gene, *Cd11c*, decreased in response to the PPE (Figure 4F), whereas gene expression of M2-like macrophage markers, including *Arg1*, *Cd206*, *Il-10*, and *Cd163* increased in response to the PPE (Figure 4F). These results suggest that the PPE caused a shift to an M2-dominant macrophage phenotype, which contributed to the attenuation of CL diet-induced inflammation.

### The PPE directly regulates M1/M2 polarization in peritoneal macrophages and inflammatory signaling in primary hepatocytes

**Figure 5:**
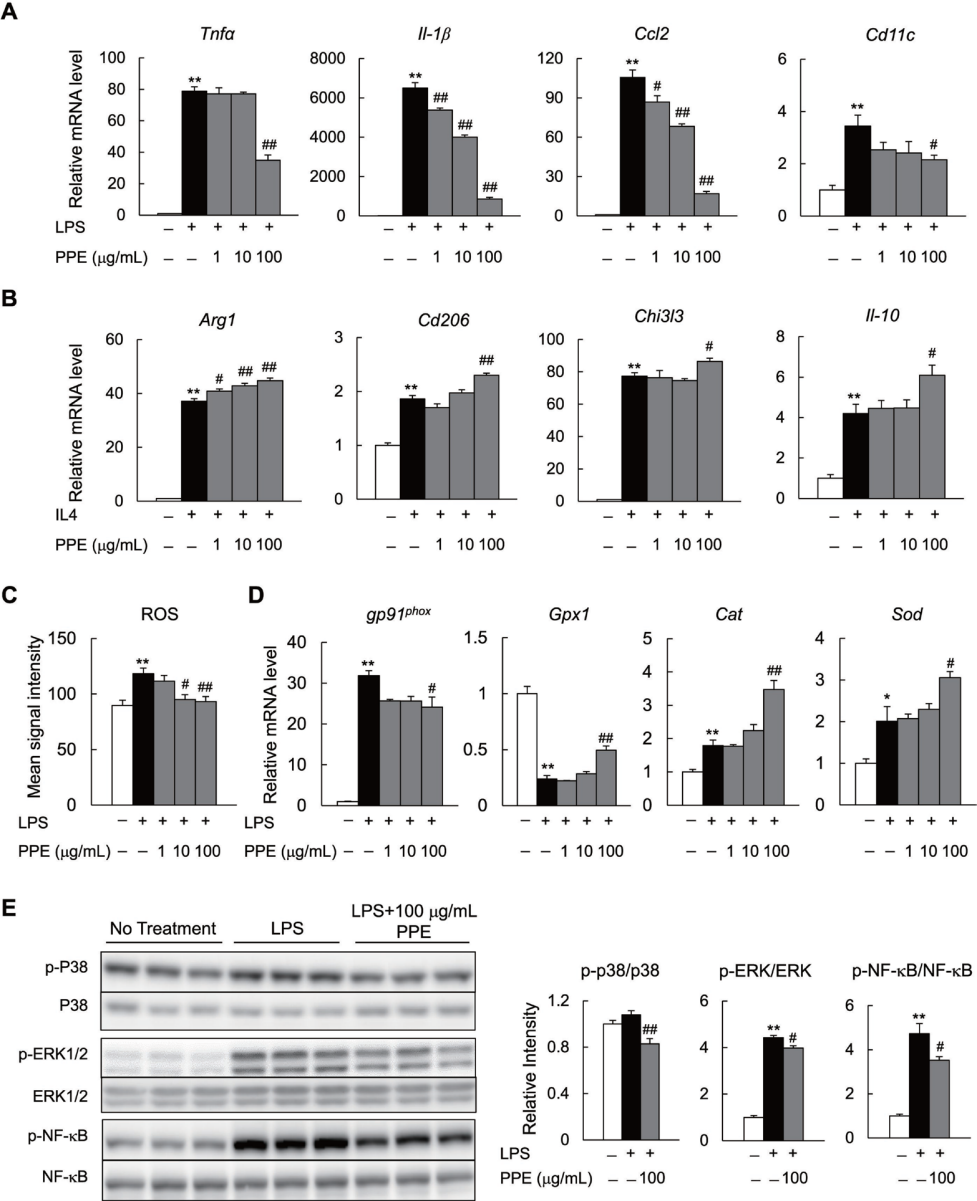
PPE directly inhibits activation of M1 macrophages and enhances M2 macrophage activation *in vitro* (**A**) mRNA expression of lipopolysaccharide (LPS)-induced M1 markers in peritoneal macrophages. (**B**) mRNA expression of IL-4-induced M2 markers in peritoneal macrophages. (**C**) Reactive oxygen species (ROS) production in LPS-treated peritoneal macrophages. (**D**) mRNA expression of NADPH oxidase subunit and anti-oxidative stress-related genes in LPS-treated peritoneal macrophages. (**E**) Immunoblot of p-p38MAPK, p-ERK, and p-NF-κB in LPS-treated Raw264.7 macrophages. *n* = 6. ^*^P < 0.05, ^**^P < 0.01, vs. no treatment cells. ^#^*P* < 0.05, ^##^*P* < 0.01, vs. LPS- and IL4-treated cells.

To test whether the PPE suppresses M1 polarization, but facilitates M2 polarization *in vitro*, we treated isolated peritoneal macrophages from C57BL/B6 mice with the PPE. PPE decreased the expression of *CD11c*, inflammatory cytokines (*Tnf-α* and *Il-1β*), and chemokine (*Ccl2*) in lipopolysaccharide (LPS)-stimulated macrophages (Figure 5A). In contrast, the PPE enhanced expression of the M2 marker genes (*Arg1, Cd206, Chi3l3,* and *IL-10*) in IL 4-stimulated macrophages (Figure 5B). LPS-mediated reactive oxygen species (ROS) production causes M1 polarization and suppresses M2 polarization in macrophages through activation of the mitogen-activated protein kinase (MAPK) and NF-κB pathways [28–30]. We determined that the increase in ROS generation in macrophages was diminished in the presence of the PPE (Figure 5C). These findings were associated with decreased mRNA expression of the NADPH oxidase subunits and increased mRNA expression of antioxidative stress genes in LPS-stimulated macrophages (Figure 5D). Moreover, the PPE significantly suppressed LPS-induced phosphorylation of MAPK (p38 and Erk) and NF-κB p65 in mice RAW264.7 macrophages (Figure 5E). The PPE also decreased expression of *Tnf-α*, *Il-1β*, *Ccl2*, and *Ccl5* in LPS- or palmitate-stimulated primary hepatocytes (Supplementary Figure 2A and 2B). Additionally, the PPE significantly suppressed palmitate-induced phosphorylation of p38 MAPK and ERK (Supplementary Figure 2C). These findings in peritoneal macrophages and primary hepatocytes indicate that the PPE may alleviate inflammatory signaling through a direct cell-autonomous mechanism.

### The PPE attenuates hepatic fibrosis in NASH mice and suppresses TGFβ signaling in a rat stellate cell line

**Figure 6:**
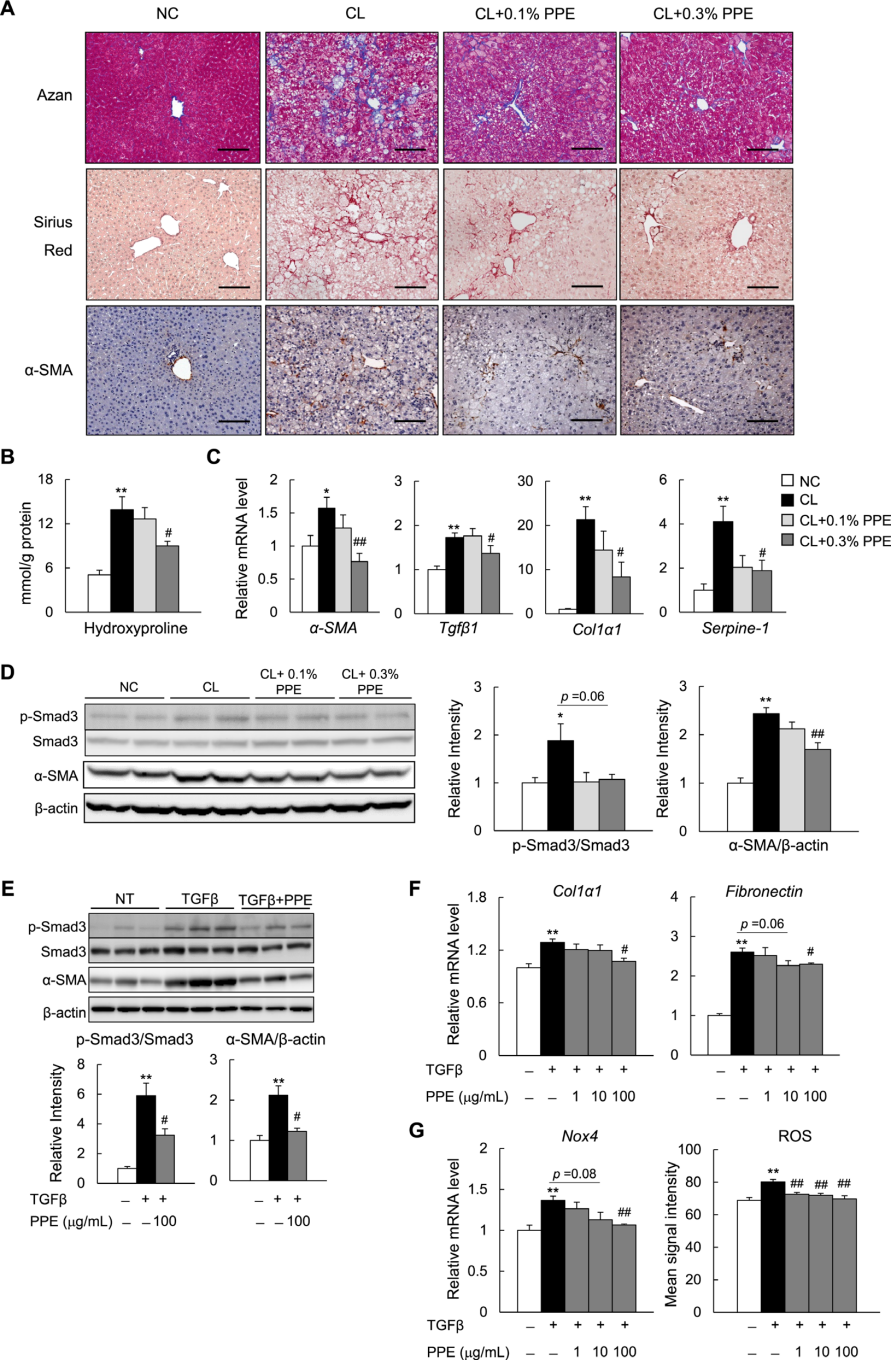
PPE attenuates hepatic fibrosis in NASH mice (**A**) Representative Azan, Sirius Red, and α-SMA immunostained liver sections. Scale bars = 100 μm. (**B**) Hydroxyproline contents in the liver. (**C**) mRNA expression of fibrogenic genes in the liver. (**D**) Immunoblot and quantification of p-Smad3 and α-SMA in the liver. *n* = 6–8. ^*^P < 0.05, ^**^P < 0.01 vs. NC diet-fed mice. ^#^*P* < 0.05, ^##^*P* < 0.01, vs. CL diet-fed mice. (**E**) Immunoblot and quantification of p-Smad3 and α-SMA in TGFβ-treated RI-T cells. (**F**) mRNA expression of fibrogenic genes in TGFβ1-treated RI-T cells. (**G**) mRNA expression of *Nox4* and ROS production in TGFβ-treated RI-T cells. *n* = 6. ^*^P < 0.05, ^**^P < 0.01, vs. no treatment cells. ^#^*P* < 0.05, ^##^*P* < 0.01, vs. TGFβ-treated cells.

Next, we determined the effects of the PPE on hepatic fibrosis in NASH mice. Histological analyses with Sirius Red and Azan staining revealed that hepatic fibrosis was induced by the CL diet, and attenuated by supplementation with 0.3% PPE (Figure 6A). Consistent with the histological findings, the PPE reduced hepatic hydroxyproline, a marker of collagen fiber content (Figure 6B). Moreover, the number of α-smooth muscle actin (SMA)-positive activated HSCs, a major fibrogenic cell, increased in response to the CL diet, and decreased in response to the PPE (Figure 6A). The decrease in number of α-SMA^+^ cells caused by the PPE was further verified by the results of quantitative real-time polymerase chain reaction (PCR) (Figure 6C) and immunoblotting (Figure 6D). Additionally, compared with the NC diet, the CL diet increased mRNA expression of fibrogenic genes, including *Tgfβ1*, collagen type I, alpha 1 (*Col1α1*), and plasminogen activator inhibitor-1 (*Serpine-1*) (Figure 6C). In contrast, PPE administration decreased the expression of these fibrogenic genes (Figure 6C). Smad3 is a key transcription factor required for TGFβ-induced extracellular matrix synthesis-activated HSCs [31–33]. The PPE tended to decrease CL diet-induced phosphorylation of Smad3, and significantly decreased α-SMA protein levels in the liver (Figure 6D). To examine whether the PPE directly suppressed activation of stellate cells, we assessed the effect of the PPE on TGFβ signaling in the RI-T rat stellate cell line. The PPE decreased TGFβ-induced phosphorylation of Smad3 and α-SMA protein levels in RI-T cells (Figure 6E). Consistently, the PPE reduced mRNA expression of TGFβ-induced fibrogenic genes (*Col1a1* and *fibronectin*) (Figure 6F). Moreover, the PPE decreased mRNA expression of *Nox4* and intracellular ROS levels in TGFβ-stimulated RI-T cells (Figure 6G).

## DISCUSSION

In the current study, we demonstrated that the PPE mitigated hepatic steatosis, oxidative stress, insulin resistance, hepatic inflammation, and fibrosis in diet-induced NASH mice. In addition, our *in vitro* studies suggest that the PPE may mitigate NASH by acting on both parenchymal and non-parenchymal cells of the liver. First, the PPE treatment decreased lipid accumulation, accompanied by decreased expression of lipogenic genes and increased expression of fatty acid oxidation genes in oleate-loaded primary hepatocytes. Second, the PPE suppressed M1-associated inflammatory signaling, gene expression, and ROS production in LPS-stimulated peritoneal macrophages. Lastly, the PPE decreased TGFβ-induced phosphorylation of Smad3 and α-SMA protein levels, as well as intracellular ROS levels in RI-T rat stellate cells. These results suggest that the PPE mitigates lipid accumulation in hepatocytes and suppresses inflammatory activation of hepatic macrophages and stellate cells, thereby preventing the progression of NASH.

Our results indicate that the beneficial effects of the PPE on hepatic steatosis and insulin sensitivity were not secondary to the reduction in body mass and food intake. Instead, the PPE mitigated CL diet-induced oxidative stress and inflammation in the liver. Hepatic inflammation mediated by M1-like liver macrophage-derived ROS, cytokines, and chemokines in NASH promotes lipogenesis by inhibiting insulin signaling and activation of SREBP [25, 34, 35]. In contrast, M2-like macrophages, which arise from stimulation by T helper type 2 (Th2) cytokines (IL4/13), secrete various anti-inflammatory molecules (IL10) [36]. Accumulating evidence has established that the development of NASH is strongly influenced by an imbalance in the ratio between M1 and M2 macrophages, leading to insulin resistance and chronic inflammation [37]. In fact, specific ablation of M1-like macrophages restores insulin sensitivity and liver lipid levels in diet-induced obese mice [38], while deleting Pparδ, which promotes M2 polarization, thereby predisposing lean mice to develop insulin resistance [39]. Therefore, M2-dominant polarization of hepatic macrophages accounts, at least in part, for the protection against insulin resistance, hepatic steatosis, and inflammation in PPE-supplemented NASH model mice.

M1-polarized liver macrophages dominantly produce TGFβ1, a key fibrogenic cytokine, which facilitates transdifferentiation of quiescent HSCs into highly proliferative myofibroblast-like activated HSCs, which are pivotal effectors in hepatic fibrogenesis [40]. In addition, TGFβ induces NOX4 following ROS production during HSC activation, which is required to activate hepatic myofibroblasts and express Smad3 [41–43]. We showed that PPE supplementation suppressed activation of HSCs, and decreased expression of TGFβ1 in the liver of NASH mice. Additionally, PPE suppressed TGFβ signaling and decreased intracellular ROS levels, accompanied by decreased expression of NOX4 in RI-T cells. Taken together, the PPE may alleviate lipotoxicity-induced hepatic fibrosis by suppressing TGFβ-mediated activation of HSCs, in addition to reducing inflammation.

It remains unclear as to which small molecule(s) in the PPE ameliorate diet-induced NASH. The PPE worked as an antioxidant *in vitro* and *in vivo*, and inhibited and activated the expression of genes encoding oxidation-promoting and anti-oxidative molecules, respectively. It has been reported that some small molecules, such as uracil, tyrosine, phenylalanine, and tryptophan, might act as antioxidants in the liver when placental extracts are administered orally or intramuscularly to rodents [20, 21, 44]. Moreover, the PPE is reported to induce genes encoding antioxidative enzymes (SOD and catalase) in the B16 mice melanoma cell line [45], although the molecules responsible have not been identified. However, vitamin C and vitamin E isolated from human placenta extracts, show protective effect of cutaneous oxidative stress [46]. In addition, trans-4-L-hydroxyprolyl-L-serine and cyclo-trans-4-L-hydroxyprolyl-L-serine, small peptides isolated from a human placental extract, exhibit potent antihepatitis activity in a-naphthylisothiocyanate-intoxicated rats after their oral administration [47]. Taken together, we speculate that multiple molecules, both known and unknown, together give rise to the anti-NASH effect of the PPE *in vivo*. Future studies are warranted to fully determine the molecules that are responsible in the PPE.

In conclusion, our findings show beneficial effects of a placental extract in preventing a spectrum of NASH-induced hepatic abnormalities, by suppressing excessive lipid accumulation and fatty acid peroxidation. The beneficial effects of the PPE are, at least in part, due to the M2-dominant shift in liver macrophages, and to inhibited HSC activation. Thus, the PPE has the potential to prevent the development of NASH.

## MATERIALS AND METHODS

### Porcine placental extract

The PPE used in this study was produced by Snowden Co. Ltd. (Tokyo, Japan) by combining fermentation and proteolysis. In brief, porcine placenta was obtained as afterbirth and was fermented with yeast (*Zygosaccharomyces* sp.) and lactic acid bacteria (*Pediococcus* sp.) for 1 day. After the supernatant was collected, the residual material was subjected to proteolysis to obtain the hydrolyzed supernatant. The supernatants were combined and lyophilized to afford the PPE powder. We chose this type of PPE for this study based on preliminary results showing that it was more effective than that produced solely by proteolysis in inhibiting LPS-induced expression of mRNAs encoding inflammatory cytokines (TNFα and IL-1β) in mouse peritoneal macrophages (data not shown).

### Mice and diets

Eight-week-old male C57BL/6J mice (Charles River Laboratories, Yokohama, Japan) were divided into four groups and fed for 15 weeks as follows: (1) Normal chow with 10% of calories from fat (NC, CRF-1; Charles River); (2) high-fat, high-cholesterol, and cholate diet (CL; 60% of calories from fat, 1.25% cholesterol, 0.5% sodium cholate; Research Diets, Cincinnati, OH, USA); (3) CL diet with 0.1% PPE (CL + 0.1% PPE); and (4) CL diet with 0.3% PPE (CL + 0.3% PPE). All mice were maintained on a 12 h/12-h light/dark cycle and given free access to food and water. All animal procedures were performed in accordance with the standards set forth in the Guidelines for the Care and Use of Laboratory Animals of Kanazawa University, Japan.

### Cell lines

RAW264.7 (TIB-71; ATCC, Manassas, VA, USA) cells, a murine monocytic cell line, were cultured in Dulbecco’s modified Eagle’s medium (DMEM; Invitrogen, Carlsbad, CA, USA) supplemented with 10% fetal bovine serum (FBS; Invitrogen) in a humidified atmosphere of 5% CO_2_ at 37°C until the cells reached 90% confluence. The cells were then serum-starved for 6 hours and incubated with 1 μg/mL LPS (Sigma-Aldrich, St. Louis, MO, USA) in the presence of 0, 1, 10, or 100 µg/mL PPE for 16 h. The LPS-induced inflammatory signals were examined by western blotting.

To investigate the protective effect of the PPE on fibrogenesis *in vitro*, RI-T cells (JCRB1088; JCRB Cell Bank, Osaka, Japan) were grown in RPMI1640 medium (Invitrogen) supplemented with 10% FBS (Invitrogen) in a humidified atmosphere of 5% CO_2_ at 37°C until the cells reached 90% confluence. After serum-starving the cells for 6 h, they were incubated with 3 ng/mL TGFβ (R&D Systems, Minneapolis, MN, USA) in the presence of 0, 1, 10, or 100 µg/mL PPE for 16 h.

### Biochemical analyses

Plasma TG, TC, NEFA, ALT, AST, insulin, IL-6, and CCL2 levels, as well as the hepatic content of TG, TC, NEFA, and TBARS, were measured as described previously [13, 48]. All hepatic lipids levels were normalized to liver protein levels.

### Glucose tolerance test and insulin signaling *in vivo*

Overnight-fasted mice were intraperitoneally injected with 2 g/kg body weight D-glucose for the GTT, and blood glucose was measured before, and 30, 60, 90, and 120 min after the injection. Anesthetized mice were injected in the tail vein with 5 U/kg body weight human insulin (Eli Lilly, Kobe, Japan) for 7 min to detect insulin signaling *in vivo*. Then, the mice were killed and the tissues harvested.

### Histological examination and immunohistochemistry

Paraffin wax-embedded liver sections were stained with hematoxylin and eosin, Sirius Red, Azan and immunohistochemically for F4/80 or α-SMA, as described previously [13, 49].

### Hydroxyproline assay

To assess liver collagen content, hepatic hydroxyproline content was measured by a spectrophotometric assay, as described previously [50]. Briefly, liver tissue was homogenized in ice-cold saline (1 mL), and the homogenates were further incubated on ice for 30 min with 125 mL of 50% trichloroacetic acid. Subsequently, the precipitated pellets were hydrolyzed for 24 h at 110°C in 6 N HCl, filtered, and neutralized with 10 N NaOH. The hydrolysates were oxidized with chloramine-T (Sigma-Aldrich) for 25 min. The reaction mixture was incubated in Ehrlich’s perchloric acid solution at 65°C for 20 min. Absorbance values were measured at 560 nm after cooling the samples to room temperature. Hydroxyproline content was normalized to the liver protein level.

### Quantitative real-time PCR

Quantitative real-time PCR was performed on a CFX384 (Bio-Rad, Hercules, CA, USA) using the SYBR Green Master Mix (Takara, Shiga, Japan), as described previously [49]. Primers used in the real-time PCR are shown in Supplementary Table 1.

### Immunoblots

Tissues were homogenized and sonicated in RIPA lysis buffer (Millipore, Billerica, MA, USA), supplemented with protease and phosphatase inhibitors (Roche Diagnostics, Indianapolis, IN, USA). Proteins were resolved by sodium dodecyl sulfate-polyacrylamide gel electrophoresis and transferred to polyvinylidene fluoride membranes. An immunoblot of the lysates was performed with primary antibodies (Supplemental Table 2), followed by appropriate secondary antibodies, and the proteins were visualized using chemiluminescence (Bio-Rad). Pixel intensities of the immunoreactive bands were quantified using ImageQuant TL (GE Healthcare Life Sciences, Tokyo, Japan).

### Fluorescence-activated cell sorting analysis

Isolation and preparation of liver cells were described previously [49, 51]. The isolated cells were incubated with Fc-Block (BD Bioscience, San Jose, CA, USA), followed by incubation with fluorochrome-conjugated antibodies (Supplementary Table 3). Flow cytometry was performed using a FACSAria II (BD Bioscience), and data were analyzed using the FlowJo software (Tree Star, Ashland, OR, USA).

### Isolation of peritoneal macrophages and primary hepatocytes and treatment

Mouse peritoneal macrophages and primary hepatocytes were isolated from a male C57BL/6J mouse (8–12 weeks old), as described previously [51, 52]. After culturing in DMEM (Invitrogen) without FBS for 6 h, primary hepatocytes were treated with 400 µM oleate (Sigma-Aldrich) and the PPE (0, 1, 10, or 100 µg/mL) for 16 h. Oil red O staining was performed on primary hepatocytes to evaluate lipid content in the hepatocytes, as described previously [13]. Cellular TG levels were measured as described previously [48].

To determine the effects of the PPE on LPS- or IL-4-stimulated cells, primary hepatocytes and peritoneal macrophages were incubated with 1 μg/mL LPS (Sigma-Aldrich) or 10 ng/mL IL-4 (Sigma-Aldrich) in the presence of 0, 1, 10, or 100 µg/mL of the PPE for 16 h. The mRNA levels of M1 and M2 markers in primary hepatocytes and peritoneal macrophages were measured by RT-qPCR.

Finally, to examine the direct effect of the PPE on antioxidative stress, intracellular ROS formation was determined by the 5-(and-6)-chloromethyl-2’, 7’- dichlorodihydrofluorescein diacetateacetylester (CM-H_2_DCFDA; Invitrogen) fluorescent probe in peritoneal macrophages and RI-T cells, as described previously [51].

### Statistics

All data are presented as means ± standard error. Differences in mean values between two groups were assessed using a two-tailed Student’s *t-*test. Differences in mean values among more than two groups were determined by analysis of variance (ANOVA). If the one-way ANOVA was significant, differences between individual groups were estimated by the Bonferroni post-hoc test. All calculations were performed with SPSS software (ver. 22.0; IBM Corp., Armonk, NY, USA). *P*-values < 0.05 were considered significant.

## SUPPLEMENTARY MATERIALS FIGURES AND TABLES





## Abbreviations

ALT: Alanine aminotransferase
AST: Aspartate aminotransferase
Ccl2: Chemokine (C-C motif) ligand 2
Ccr2: Ccl2 receptor
CL: High-cholesterol and high-fat diet
Col1α: Collagen type I alpha
ER: Endoplasmic reticulum
FACS: Fluorescence-activated cell sorting
GTT: Glucose tolerance test
HSC: Hepatic stellate cell
IL: Interleukin
LPS: Lipopolysaccharide
NAFLD: Nonalcoholic fatty liver disease
NASH: Nonalcoholic steatohepatitis
NC: Normal chow diet
NEFA: Non-esterified fatty acids
PPE: Porcine placental extracts
ROS: Reactive oxygen species
Scd1: Stearoyl-CoA desaturase 1
Serpine: Serpin family E member
Sma: Smooth muscle actin
Srebf1c: Sterol regulatory element binding transcription factor 1c
TBARS: Thiobarbituric acid reactive substances
TC: Total cholesterol
TG: Triacylglycerols
Tgf: Transforming growth factor
Tnf: Tumor necrosis factor
