# Supplementary material for: A porcine placental extract prevents steatohepatitis by suppressing activation of macrophages and stellate cells in mice
Source: Oncotarget. 2018 Feb 27;9(19):15047–60. doi: 10.18632/oncotarget.24587 (PMC5871096; doi:10.18632/oncotarget.24587)
Supplement: Supplementary file 1 [file oncotarget-09-15047-s001.pdf]

A porcine placental extract prevents steatohepatitis by suppressing activation of macrophages and stellate cells in mice

SUPPLEMENTARY MATERIALS

Supplementary Table 1: Mice and rat primer sequences. See Supplementary_Table_1

Supplementary Table 2: Antibodies used in immunoblotting

Antibody	
anti-phospho-JNK	Cell signaling (#9255)
anti-JNK	Cell signaling (#9258)
anti-phospho-p38 MAPK	Cell signaling (#9211)
anti-p38 MAPK	Cell signaling (#9212)
anti-phospho-NF-κB p65	Cell signaling (#3033)
anti-NF-kB p65	Cell signaling (#3034)
anti-phospho-IR β	Cell signaling (#3021)
anti-IR β	Cell signaling (#3025)
anti-phospho-Akt	Cell signaling (#9271)
anti-Akt	Cell signaling (#9272)
anti-phospho-Erk 1/2	Cell signaling (#9101)
anti-Erk 1/2	Cell signaling (#9102)
anti-CHOP	Cell signaling (#2895)
anti-GRP78	Santa Cruz (sc13968)
anti-phosphor-Smad3	Cell signaling (#9520)
anti-Smad3	Cell signaling (#9523)
Monoclonal anti-b-actin	Sigma-Aldrich (A5441)

Supplementary Table 3: Antibodies used in FACS analysis

Antibody	
PerCP-Cyanine5.5-conjugated NK1.1	eBioscience
PerCP-Cyanine5.5-conjugated CD3	eBioscience
PerCP-Cyanine5.5-conjugated CD19	eBioscience
PerCP-Cyanine5.5-conjugated TER-119	eBioscience
allophycocyanin (APC)-eFluor 780-conjugated CD45	eBioscience
phycoerythrin (PE)-conjugated CD11c	eBioscience
eFluor 450-conjugated Ly-6G (Gr-1)	eBioscience
PE/Cy7-conjugated F4/80	Biolegend
Alexa Fluor 647-conjugated CD206	Biolegend
PE-Texas Red-conjugated CD11b	Invitrogen

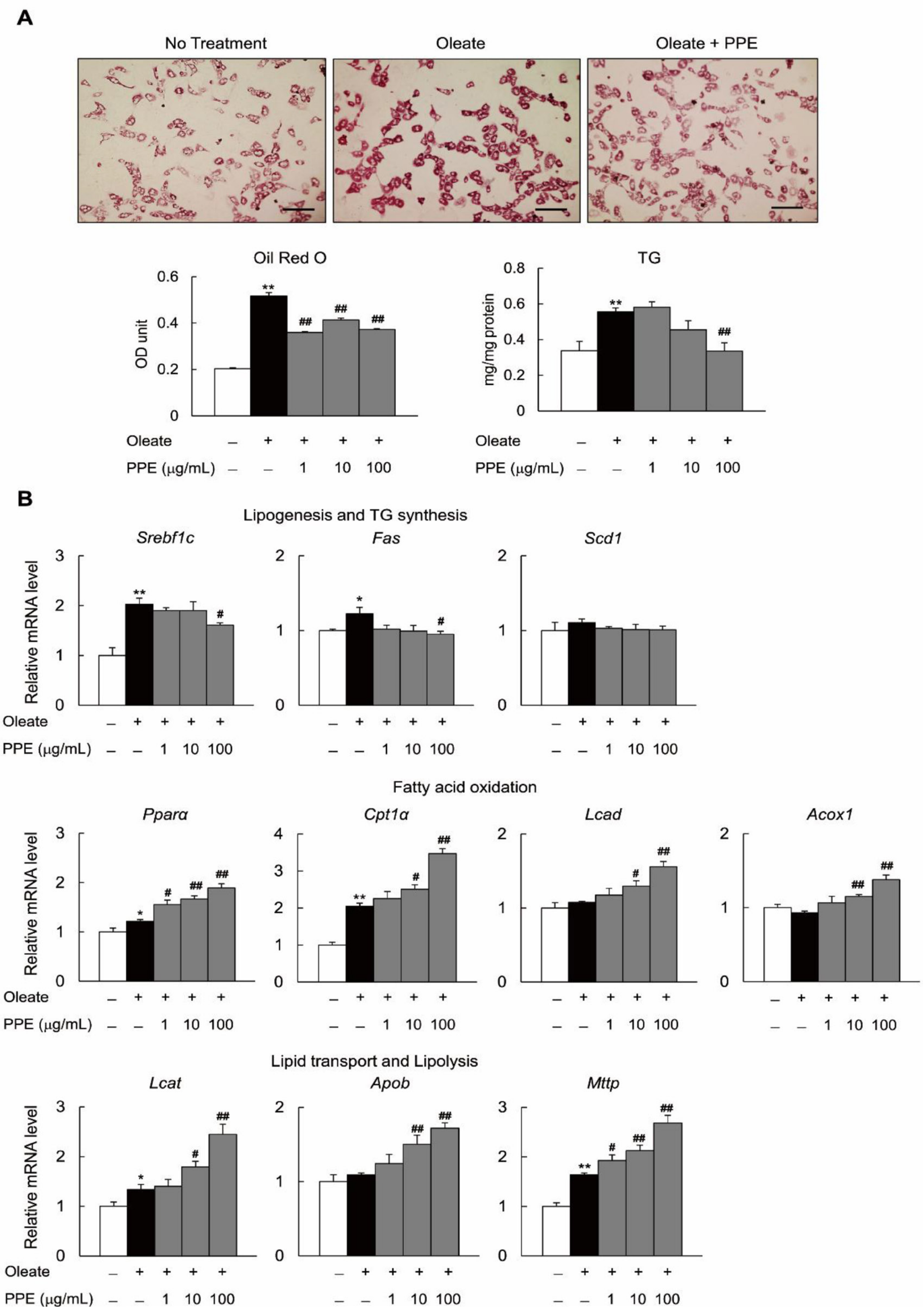

Supplementary Figure 1: Placenta extracts prevented the fat accumulation in primary hepatocytes. (**A**) Oil Red O staining of cultured primary hepatocytes and cellular TG levels. (**B**) mRNA expression of lipogenesis and lipolysis genes in the primary hepatocytes. $n = 6$. $^{*}P < 0.01$, vs. no treatment cells. $^{\#}P < 0.05$, $^{\#\#}P < 0.01$, vs. Oleate-treated cells.

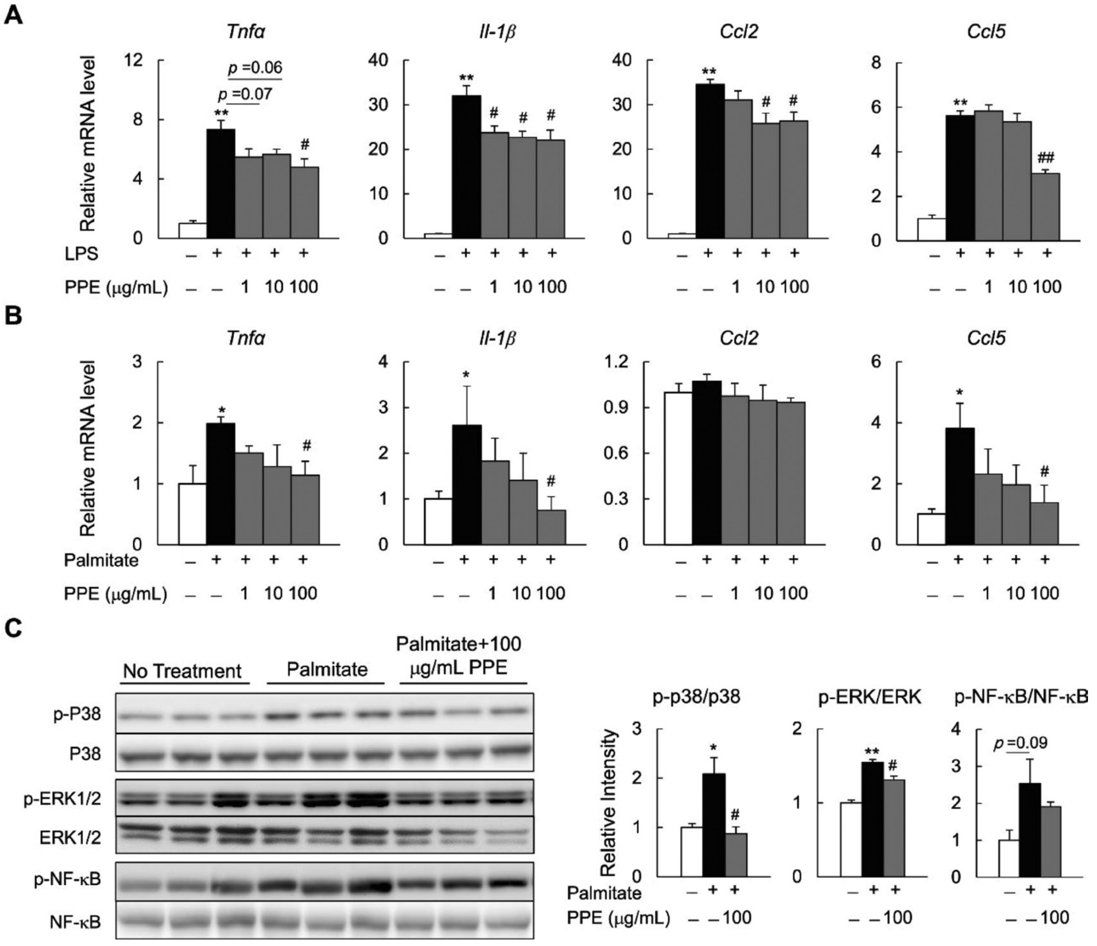

Supplementary Figure 2: Placenta extracts attenuated LPS- and palmitate-induced inflammation in primary hepatocytes. (**A**) mRNA expression of LPS-induced M1 markers in primary hepatocyte. (**B**) mRNA expression of palmitate-induced M1 markers in primary hepatocyte. (**C**) Immunoblot of p-p38MAPK, p-ERK, p-NF-κB in palmitate-treated primary hepatocytes. $n = 6$. $^{*}P < 0.05$, $^{**}P < 0.01$, vs. no treatment cells. $^{\#}P < 0.05$, $^{\#\#}P < 0.01$, vs. LPS- and palmitate-treated cells.
