# Supplementary material for: A porcine placental extract prevents steatohepatitis by suppressing activation of macrophages and stellate cells in mice
Source: Oncotarget. 2018 Feb 27;9(19):15047–60. doi: 10.18632/oncotarget.24587 (PMC5871096; doi:10.18632/oncotarget.24587)
Supplement: Supplementary file 2 [file oncotarget-09-15047-s002.docx]

**Supplementary Table 1–Mice and rat primer sequences.**

| Gene | 5’ Primer | 3’ Primer |
| --- | --- | --- |
| mAcox1 | TTATGCGCAGACAGAGATGG | AGGCATGTAACCCGTAGCAC |
| mApob | GCCCATTGTGGACAAGTTGATC | CCAGGACTTGGAGGTCTTGGA |
| mArg1 | CTCCAAGCCAAAGTCCTTAGAG | AGGAGCTGTCATTAGGGACATC |
| mα-SMA | TGTGCTGGACTCTGGAGATG | GAAGGAATAGCCACGCTCAG |
| mCat | CCAGCGACCAGATGAAGCAG | CCACTCTCTCAGGAATCCGC |
| mCcl2 | AGGTCCCTGTCATGCTTCTGG | CTGCTGCTGGTGATCCTCTTG |
| mCcr2 | ATTCTCCACACCCTGTTTCG | GATTCCTGGAAGGTGGTCAA |
| mCd11c | AAAATCTCCAACCCATGCTG | CACCACCAGGGTCTTCAAGT |
| mCd163 | GGGTCATTCAGAGGCACACTG | CTGGCTGTCCTGTCAAGGCT |
| mCd206 | CAAGGAAGGTTGGCATTTGT | CCTTTCAGTCCTTTGCAAGC |
| mCpt1α | AAACCCACCAGGCTACAGTG | TCCTTGTAATGTGCGAGCTG |
| mCol1α1 | ACGTCCTGGTGAAGTTGG TC | CAG GGA AGC CTC TTT CTC CT |
| mF4/80 | CTTTGGCTATGGGCTTCCAGTC | GCAAGGAGGACAGAGTTTATCGTG |
| mFas | AGAGACGTGTCACTCCTGGACTT | GCTGCGGAAACTTCAGAAAAT |
| mGp^91phox^ | TTGGGTCAGCACTGGCTCTG | TGGCGGTGTGCAGTGCTATC |
| mGpx1 | TTCGGACACCAGGAGAATGG | TAAAGAGCGGGTGAGCCTTC |
| mIl-1β | CTGAACTCAACTGTGAAATGCCA | AAAGGTTTGGAAGCAGCCCT |
| mIl-10 | GCTCTTACTGACTGGCATGAG | CGCAGCTCTAGGAGCATGTG |
| mLcad | TCACCACACAGAATGGGAGA | ACGCTTGCTCTTCCCAAGTA |
| mLcat | GCTCTGTGGCCAGTGGCAGG | AGGAGTGCGGTAGGCACCCA |
| mMttp | GCC CTA GTC AGG AAG CTG TG | CCA GCA GGT ACA TTG TGG TG |
| mNox4 | GGATCACAGAAGGTCCCTAGCAG | GCAGCTACATGCACCTGAGAA |
| mP^22phox^ | GTCCACCATGGAGCGATGTG | CAATGGCCAAGCAGACGGTC |
| mP^40phox^ | GCCGCTATCGCCAGTTCTAC | GCAGGCTCAGGAGGTTCTTC |
| mP^47phox^ | GATGTTCCCCATTGAGGCCG | GTTTCAGGTCATCAGGCCGC |
| mP^67phox^ | CTGGCTGAGGCCATCAGACT | AGGCCACTGCAGAGTGCTTG |
| mPparα | GAGGGTTGAGCTCAGTCA GG | GGTCACCTACGAGTGGCATT |
| mScd1 | CATCATTCTCATGGTCCTGCT | CCCAGTCGTACACGTCATTTT |
| mSerpin-1 | TCAGCCCTTGCTTGCCTCAT | TCAGCCCTTGCTTGCCTCAT |
| mSod | CAGCATGGGTTCCACGTCCA | CACATTGGCCACACCGTCCT |
| mSrebf-1c | GGAGCCATGGATTGCACATT | GGCCCGGGAAGTCACTGT |
| mTgfβ1 | TGAGTGGCTGTCTTTTGACG | TCTCTGTGGAGCTGAAGCAA |
| mTnfα | AAGCCTGTAGCCCACGTCGTA | GGCACCACTAGTTGGTTGTCTTTG |
| mβ-actin | AGGCCCAGAGCAAGAGAGGTA | GGGGTGTTGAAGGTCTCAAACA |
| m18S | AGG CCC AGA GCA AGA GAG GTA | GGG GTG TTG AAG GTC TCA AAC A |
| rCol1α1 | GAGTGAGGCCACGCATGA | AGCCGGAGGTCCACAAAG |
| rFibronectin | CAGCAGCAAGCCAGTTTCCA | TGAAGGCAGCCACCTGACAC |
| rNox4 | GGATCACAGAAGGTCCCTAGC | AGAAGTTCAGGGCGTTCACC |
| rGAPDH | GGCAAGTTCAATGGCACA | AGCACCAGCATCACCCCATTT |
